# Effects of plant-based diets combined with exercise training on leptin and adiponectin levels in adults with or without chronic diseases: a systematic review and meta-analysis of clinical studies

**DOI:** 10.3389/fnut.2024.1465378

**Published:** 2024-10-09

**Authors:** Fatemeh Kazeminasab, Rouholah Fatemi, Reza Bagheri, Heitor O. Santos, Fred Dutheil

**Affiliations:** ^1^Department of Physical Education and Sports Science, Faculty of Humanities, University of Kashan, Kashan, Iran; ^2^Department of Sport Physiology, Dehdasht Branch, Islamic Azad University, Dehdasht, Iran; ^3^Department of Exercise Physiology, University of Isfahan, Isfahan, Iran; ^4^School of Medicine, Federal University of Uberlandia (UFU), Uberlandia, Minas Gerais, Brazil; ^5^Université Clermont Auvergne, CNRS, LaPSCo, Physiological and Psychosocial Stress, CHU Clermont-Ferrand, University Hospital of Clermont-Ferrand, Preventive and Occupational Medicine, Witty Fit, Clermont-Ferrand, France

**Keywords:** metabolic syndrome, adipokines, leptin, metabolic health, obesity

## Abstract

**Background:**

The effects of exercise training combined with plant-based diets (PBD) on leptin and adiponectin levels have been studied. However, little is known regarding the impact of exercise training combined with PBD on leptin and adiponectin levels in adults with or without chronic diseases.

**Methods:**

PubMed, Web of Science, and Scopus were searched to identify original articles, published until May 2024, to assess the effects of exercise training combined with PBD on leptin and adiponectin levels in adults with or without chronic diseases. Standardized mean differences (SMD) and 95% confidence intervals were calculated using random models.

**Results:**

Nine studies comprising 960 participants with overweight and obesity were included in the current meta-analysis. Exercise training combined with PBD reduced leptin [SMD = -0.33 (95% CI: −0.62 to −0.04); *p* = 0.025] while increasing adiponectin [SMD = 0.93 (95% CI: 0.12 to 1.74); *p* = 0.024] levels.

**Conclusion:**

Exercise training combined with PBD is suggested as a non-invasive intervention for reducing leptin while increasing adiponectin levels to control body mass and other disorders related to obesity in adults.

## 1 Introduction

Obesity is a global phenomenon that has reached epidemic proportions, affecting both developed and developing countries (1). The worldwide prevalence of obesity is escalating swiftly, with a substantial surge in the past years (2). Obesity is associated with several metabolic disorders and chronic diseases, including type 2 diabetes mellitus (T2DM), cardiovascular diseases (CVD), and certain types of cancer (3, 4). Adipose tissue plays a crucial role in the progression of obesity and its associated health risks (4). Leptin and adiponectin are adipokines that modulate energy balance, cardiometabolism, and low-grade inflammation (5). Leptin levels are elevated in individuals with obesity and are associated with leptin resistance, which is related to various metabolic diseases (6). Leptin affects body mass by reducing food intake and increasing energy intake (7). It also plays a role in the reproductive processes, angiogenesis, blood pressure, glucose and lipid metabolism, bone formation, and immune responses (8–12). Leptin levels are positively correlated with fat mass, resulting in elevated levels in individuals with obesity (13). On the other hand, adiponectin regulates energy expenditure, stimulates appetite, and its secretion escalates in tandem with enhanced insulin sensitivity (6). Adiponectin, the adipokine most abundantly secreted by adipose tissue (7), exhibits a decline in serum concentrations with obesity and is also associated with an elevated risk of T2DM and CVD (14, 15).

Drug and surgical interventions (e.g., bariatric surgery or intragastric balloon) for obesity, despite demonstrating effectiveness, frequently entail a multitude of adverse effects and the recurrence of body mass (16–18). Consequently, efforts must be directed toward dietary and lifestyle modifications, especially those that are abundant in plant-based diets (PBD), since those consuming PBD lower their risk for obesity, CVD, T2DM, and other health conditions (19, 20). PBD are capable of potentially exerting an influence on the quantities of leptin and adiponectin in the human body (21, 22). As per the investigation conducted by Lederer et al. (23), plasma adiponectin concentrations were noticeably higher in females who transitioned from an omnivorous to PBD when compared to those following a diet rich in meat; nevertheless, this study did not observe any significant alterations in plasma leptin concentrations. Another study conducted by Menzel et al. (24) failed to identify any notable variances in inflammatory markers, including adiponectin, between vegans and individuals who consume both plant-and animal-based foods.

While vegetarian and vegan diets have the potential to influence leptin and adiponectin levels, further research is needed to fully understand the impacts of PBD. This includes exploring the benefits of reducing animal food consumption rather than completely eliminating it. PBD focus on foods primarily from plants; however, they are not the same as vegetarian or vegan diets since PBD may include meat and dairy, although proportionately predominating in plant sources such as fruits, vegetables, nuts, seeds, oils, whole grains, legumes, and beans (25). However, it is widely acknowledged that the Mediterranean diet and other vegetarian diets (e.g., semi-vegetarian, flexitarian, pescatarian, lacto-ovo vegetarian, and vegan) are often classified as PBD (26).

Many investigations have examined the correlation between physical activity and leptin and adiponectin levels. A narrative review reported that physical activity (only daily physical activity was measured but not any specific physical exercises) elevated adiponectin and decreased leptin levels while simultaneously lowering body mass index (BMI) and body fat percentage (BFP) (27). Physical activity, either on its own or in conjunction with changes in diet or lifestyle, can reduce the levels of leptin in individuals with prediabetes (19) and obesity (28). In a meta-analysis of seven studies by Jadhav et al. (29), physical activity promotion, with or without dietary or lifestyle modification, reduced leptin levels but had no remarkable effects on adiponectin levels in individuals with prediabetes. Seemingly, there is a weak to moderate relationship between aerobic exercise with increased adiponectin levels and decreased leptin levels (30), but it cannot be neglected and ought to be further examined in conjunction with diet, mainly PBD as a cost-effective, low-risk intervention that may improve obesity and cardiometabolic parameters (31, 32). In terms of different forms of physical activity, it was shown that only aerobic exercise but not other forms led to a rise in adiponectin levels and a decrease in leptin levels. To summarize, engaging in physical activity, particularly aerobic exercise, results in increased adiponectin levels and decreased leptin levels in individuals with prediabetes and diabetes (30).

The combination of both exercise training and PBD would be anticipated to produce an additive effect, which may lead to improved health outcomes after the intervention (33–35). The previous study suggests that a combination of exercise training with the Mediterranean diet is more effective than a normal diet for reducing leptin in overweight/obese adults with metabolic syndrome (36). Nevertheless, these results are not consistently reliable. For example, one study did not suggest further increases in adiponectin with exercise training in combination with Mediterranean diet as compared with non-vegetarian diets (37). However, the combined impact of exercise training combined with PBD has not been elucidated in previous meta-analyses. Therefore, the aim of this study was to determine the impact of exercise training combined with PBD on leptin and adiponectin in adults with or without chronic diseases using a systematic review and meta-analysis method.

## 2 Methods

### 2.1 Study registration

The current systematic review and meta-analysis was conducted according to the Preferred Reporting Items for Systematic Reviews and Meta-Analyses (PRISMA) 2020 guidelines (38) and the Cochrane Handbook of Systematic Reviews of Interventions. The systematic review and meta-analysis was registered prospectively with the International Prospective Register of Systematic Reviews (PROSPERO) under the registration number: CRD42023457202.

### 2.2 Search strategy

PubMed, Scopus, and Web of Science were the primary databases searched to recognize original research articles, published up until May 2024, using a combination of exercise training and PBD terms and the outcomes (leptin and adiponectin). In addition, reference lists of all included studies and previous relevant meta-analyses (39, 40) were searched to detect records not found during the initial electronic search. The search was limited to articles written in the English language and human studies. There was no limit on publication dates. Studies were limited only based on the age of the participants. Adults were included in the present study. Studies performing PBD combined with exercise training interventions were searched by using the following keywords: “vegetarian diet,” “diet,” “Mediterranean diet,” “vegetarian diet,” “plant-based diet,” “paleolithic diet,” “dietary pattern,” “DASH,” “vegan diet,” “lacto-ovo-vegetarian diet,” and “exercise,” “training,” “exercise training,” “physical activity,” and “adipokine,” “adipocytokine,” “adiponectin,” “leptin.” The search strategy for each database is reported in Supplementary Table S1.

### 2.3 Eligibility criteria

Studies were eligible for inclusion if they met the following PICO (population, intervention, comparison, and outcome) criteria: (1) For population, adults with or without chronic diseases. (2) For the intervention, studies with exercise training combined with PBD were included. (3) For comparison, the pre- and post-test comparisons for the combined effects of exercise training combined with PBD were required. (4) For outcomes, studies that reported leptin and adiponectin measured using a fully validated method were included.

### 2.4 Study selection

**Figure 1:**
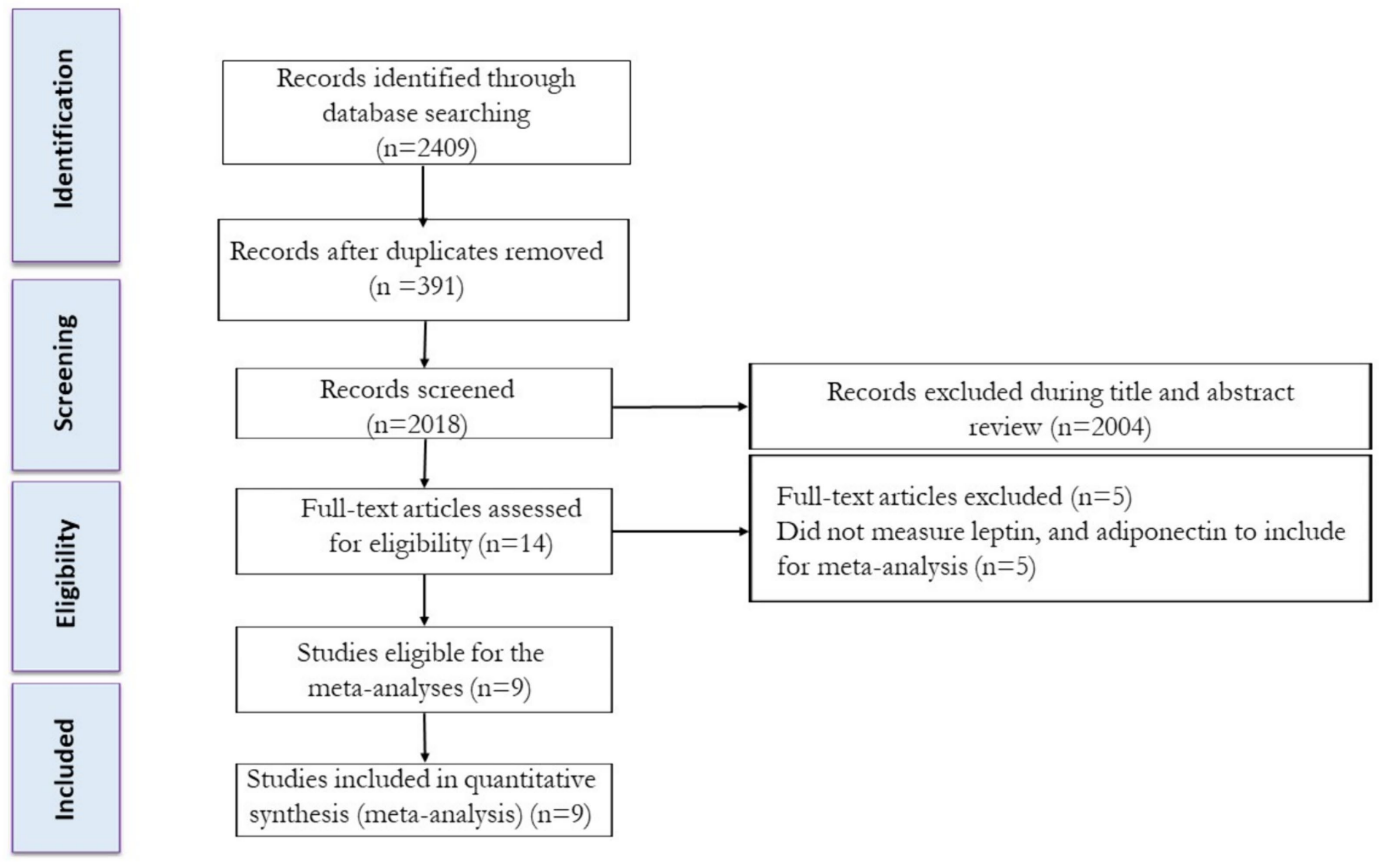
Flow diagram of systematic literature search.

The study selection process is shown in Figure 1. Following the removal of duplicate studies, titles and abstracts of articles were independently assessed, and then the full texts of potentially eligible studies were reviewed by two reviewers to determine eligibility. Any disagreements were resolved through discussion with another author.

Studies that met the following criteria were included in this systematic review and meta-analysis: (1) randomized controlled trials (RCTs), prospective cohort studies, non-randomized controlled trials (non-RCTs); (2) the types of diets included vegetarian, lacto-ovo vegetarian, lacto-vegetarian, ovo-vegetarian diets, Mediterranean diets, Apulian diet (aphypoD); (3) exercise training types were not limited; (4) studies that examined the effect of exercise training combined with PBD on serum or plasma leptin and adiponectin; and (5) studies that presented outcome data of interest as the mean and standard deviations (SD), mean and standard error of the mean (SEM), or quartile and 95% confidence interval (CI) in the intervention group. The exclusion criteria were as follows: (1) vegetarian participants; (2) other interventions along with exercise training and diet have been applied; (3) studies that were animal studies, narrative reviews, commentaries, letters, books, case reports, case studies, pilot studies, and conferences; and (4) studies that were not published in English language.

### 2.5 Data extraction

Data extraction was completed independently by 2 researchers, and disagreements were resolved through discussion with a third review author. The following study characteristics were extracted: (A) participant information, including age, biological sex, BMI, health status, and sample size; (B) study design; and (C) exercise training characteristics (type, duration, and frequency); (D) diet characteristics and duration of intervention; and I outcome analysis. For each outcome (leptin, and adiponectin), pre-and post-intervention (mean and SD), or mean differences and associated standard deviations of the experimental group were entered into the meta-analyses to generate forest plots. If the means and standard deviations (SDs) were not reported, the SDs were calculated from standard errors of means (SEM), medians and interquartile ranges (IQRs), or means and IQRs (41–43).

### 2.6 Quality assessment

The risk of bias was evaluated using the Physiotherapy Evidence Database (PEDro) scale. Two items were omitted from the initial 11-item scale due to factors such as similarity of groups at baseline, absence of blinding for participants and intervention providers, and the inability to obscure the allocated dietary conditions during the studies for both participants and intervention providers. The scale used for the current study consisted of nine items: (1) specified eligibility criteria, (2) randomized participant allocation, (3) concealed allocation, (4) blinding of all assessors, (5) evaluated outcomes in 85% of participants, (6) intention-to-treat (ITT) analysis, (7) reporting of statistical comparisons between groups, (8) and point measures and measures of variability (Supplementary Table S2).

### 2.7 Statistical analysis

Meta-analyses were performed using the Comprehensive Meta-analysis (CMA) software (version 2.0, United States) to compute standardized mean differences (SMD) and 95% CIs for outcomes using random-effects models. Effect sizes were calculated to compare the impact of exercise training combined with PBD on leptin and adiponectin in adults. Heterogeneity was evaluated by using the I^2^ statistic, and significance was set at *p* < 0.05. According to Cochrane guidelines, I2 statistics were interpreted as follows: 25% as low, 50% as moderate, and 75% as high heterogeneity. Publication bias was detected through the interpretation of funnel plots. If publication bias was present, Egger’s test was used as a secondary test. Significant publication bias was deemed apparent if *p* < 0.1 (44). Furthermore, the trim and fill method was used to correct the potential effects of publication bias when visual interpretation of funnel plots demonstrated publication bias.

## 3 Results

### 3.1 Included studies

Based on our original search technique, we discovered a combined total of 808 articles in PubMed, 136 articles in Scopus, and 1,465 articles in Web of Science. After excluding duplicate records and evaluating the titles and abstracts, 16 studies were determined to be relevant and necessitated a comprehensive assessment of their complete texts. After conducting a detailed assessment of the full texts, five studies were excluded for the following reasons: did not measure leptin and adiponectin (*n* = 5) levels. In this systematic review and meta-analysis, a total of nine studies were evaluated, which comprised intervention groups involving the combination of exercise training and PBD. The flow diagram of the systematic literature search is shown in Figure 1.

### 3.2 Participant characteristics

A combined group of 960 adults with different diseases such as metabolic syndrome, obesity, postmenopausal women, obstructive sleep apnea, T2DM, and coronary artery disease were included, with sample sizes ranging from 37 (45) to 266 (46). The average age ranged from 18 years (34) to 75 years (36), while the BMI ranged from 24.6 kg.m^−2^ (33) to 40 kg.m^−2^ (36, 46). A total of nine studies were encompassed in the analysis. Among them, seven studies had both males and females (20, 35–37, 45–47), whereas one study only included males (34) and one study only included females (33). The participants’ health status varied across the studies, including healthy elderly participants (47), metabolic syndrome (34, 46), overweight and obesity (20, 36), postmenopausal women (33), obstructive sleep apnea (37), T2DM (45), and coronary artery disease (35). Table 1 provides a more comprehensive breakdown of the characteristics of the participants.

**Table 1:** Study, participants, and intervention characteristics.

Source, year		Study characteristics			Participant characteristics				Exercise training characteristics
	Design of study	Sample size (sex)	Intervention duration	Outcomes	Health status	Age (years)	BMI (kg.m^−2^)	Diet characteristics	
						Mean ± SD	Mean ± SD		
Bendinelli et al., 2023 (33)	Factorial randomized trial	82 F	24-month	Leptin Adiponectin	Postmenopausal women	PBD + Ex: 58.7 ± 5.3	PBD + Ex: 24.6 ± 3.2	Plant foods with a low glycemic load, low in saturated- and trans-fats and alcohol, and rich in antioxidants and fiber.	Moderate-intensity PA three MET-hours/day, up to one hour/day. Suggested daily activities were walking at moderate pace, and biking- at least one hour/week of more strenuous activity (6–10 MET)
Esposito et al., 2011 (34)	Randomized controlled trial	52 M	24-month	Adiponectin	Sedentary overweight with metabolic syndrome	PBD + Ex: ≥18	PBD + Ex: ≥26	Mediterranean regime includes fruits, vegetables, nuts, whole grains and olive oil daily (Carbohydrates 50 to 60%, proteins 15 to 20%, total fat *≤*30%, saturated fat *<*10%)	Mainly walking for a minimum of 39–156 min per day, but also swimming or aerobic ball games
Georgoulis et al., 2021 (37)	Randomized controlled clinical trial	60 (M & F)	6-month	Adiponectin	Overweight patients with moderate-to-severe obstructive sleep apnea	PBD + Ex: 48 ± 10.0	PBD + Ex: 35.8 ± 6.0	Mediterranean diet, i.e., a primarily plant-based dietary pattern (consumption of olive oil, vegetables, legumes, whole grains, fruits, and nuts, moderate consumption of poultry and fish)	≥150 min/week of moderate- and high-intensity walking
Hernando-Redondo et al., 2022 (46)	Randomized, controlled, multicenter clinical trial	266 (M & F)	12-month	Leptin	Patients with metabolic syndrome	PBD + Ex: 65.44 ± 4.62	PBD + Ex: 27–40	Mediterranean diet adherence	150 min/week 2207.52 MET min/week
Salas-Salvadó et al., 2019 (36)	Randomized controlled trial	261 (M & F)	12-month	Leptin	Adults with overweight and obesity	PBD + Ex: 55–75	PBD + Ex: 27–40	Mediterranean diet adherence extra with Virgin olive oil (1 L/month) and raw nuts (125 g/month)	Not provided
Kahleova et al., 2011 (45)	Randomized controlled trial	37 (M & F)	6-month	Adiponectin	Patients with type 2 diabetes	PBD + Ex: 54.6 ± 7.8	PBD + Ex: 35.1 ± 6.1	Vegetarian diet (0% of energy from carbohydrates, 15% protein and 25% fat) consisted of vegetables, grains, legumes, fruits and nuts	1 h, 2 times/week of individualized exercise program based on their history of physical activity with 60% MHR
Koeder et al., 2023 (47)	Non-randomized controlled trial	80 (M & F)	10-week	Adiponectin	Healthy elderly	PBD + Ex: 59.7 ± 8.36	PBD + Ex: 27.2 ± 0.5	Plant-based diet including soya foods, nuts, seeds, and healthy oils	Suggestions for recreational walking and cycle
Telles et al., 2010 (20)	Non-randomized controlled trial	47 (M & F)	1-week	Leptin	Adults with obesity	PBD + Ex: 40.3 ± 10.2	PBD + Ex: 35.97 ± 5.72	Vegetarian diet includes fresh fruits and vegetables, lentils	Yoga program consisted of 2 sessions each day.
Voeghtly et al., 2013 (35)	Non-randomized controlled trial	76 (M & F)	12-month	Leptin	Patients with coronary artery disease	PBD + Ex: 60.6 ± 7.6	PBD + Ex: 32.9 ± 7.2	Low-fat vegetarian diet (<10% of calories from fat)	180 min/week of moderate aerobic exercise on treadmill

M, male; F, female; Ex, exercise; MET, metabolic equivalent; PA, physical activity; PBD, plant-based-diets; MHR, maximum heart rate.

### 3.3 Intervention characteristics

A combination of exercise training and PBD methodologies was implemented across the included studies in the analysis. Some studies used plant foods with a low glycemic load, low in saturated fats and alcohol, and rich in antioxidants and fiber (33), PBD including soya foods, nuts, seeds, and healthy oils (47), and others used Mediterranean diet including fruits, vegetables, nuts, whole grains, and olive oil daily (34, 36, 37, 46). Additionally, the length of the interventions varied, ranging from 1 week (20) to 24 months (33–37, 45–47). The studies incorporated various forms of exercise training interventions, including moderate-intensity physical activity, moderate- and high-intensity walking, yoga programs, moderate aerobic exercise on a treadmill, swimming or aerobic ball games, or cycling. In most studies, participants exercised 150 min/week. Detailed information about the intervention characteristics can be found in Table 1.

### 3.4 Meta-analysis

#### 3.4.1 The effects of PBD combined with exercise training on leptin in adults

**Figure 2:**
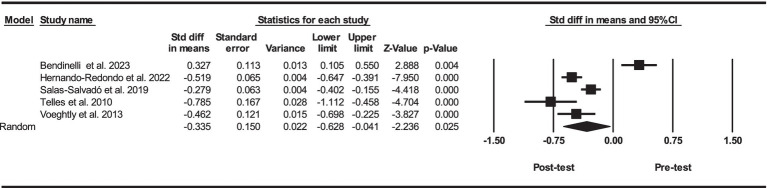
Forest plot of the effects of exercise training combined with PBD on serum leptin. Data are reported as SMD (95% confidence limits). SMD, standardized mean differences.

Based on five intervention arms, PBD combined with exercise training had significantly lower serum concentrations of leptin [SMD = -0.33 (95% CI: −0.62 to −0.04) and *p* = 0.025] (Figure 2). The studies included in the analysis demonstrated high heterogeneity (I^2^ = 92.19%, *p* = 0.001). The absence of publication bias was supported by the results of funnel plots and Egger’s test (*p* = 0.90). Given that the result of the funnel plots and Egger’s test was not significant, the trim and fill method was not performed.

#### 3.4.2 The effects of PBD combined with exercise training on adiponectin in adults

**Figure 3:**
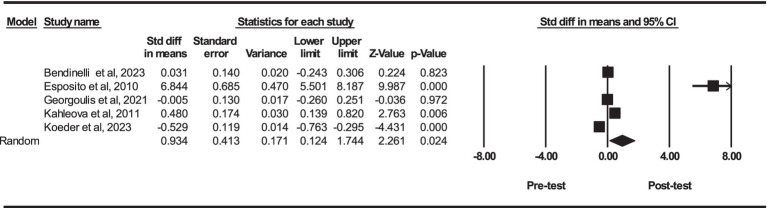
Forest plot of the effects of exercise training combined with PBD on serum adiponectin. Data are reported as SMD (95% confidence limits). SMD, standardized mean differences.

Based on five intervention arms, PBD combined with exercise training had significantly higher serum concentrations of adiponectin [SMD = 0.93 (95% CI: 0.12 to 1.74) and *p* = 0.024] (Figure 3). The studies included in the analysis demonstrated high heterogeneity (I^2^ = 96.84%, *p* = 0.001). The existence of publication bias was supported by the results of funnel plots and Egger’s test (*p* = 0.003). In addition, the trim and fill results showed that the overall changes were as follows: adiponectin [SMD = 0.93 (95% CI: 0.12 to 1.74)]. The results of adiponectin did not change [SMD = 0.93 (95% CI: 0.12 to 1.74)]. Since no bias is present in these simulations all “missing” studies detected are because of chance funnel plot asymmetry.

### 3.5 Quality assessment

The PEDro tool was used to assess the methodological quality of each individual study, and their scores varied between 3 and 9 out of a possible maximum of 9 points. One study had a score of 9 (37), one study had a score of 8 (33), two studies had scores of 6 (36, 45), four studies had 5 (20, 34, 35, 46), and one study had a score of 3 (47). Most of the PEDro scores were lowered due to three items (concealed allocation, blinding of all assessors, and intention-to-treat analysis). The details of the quality analysis are shown in Supplementary Table S2.

## 4 Discussion

The aim of this meta-analysis was to investigate the effect of PBD combined with exercise training on leptin and adiponectin levels in adults with or without chronic diseases. The main findings of the study showed that exercise training combined with PBD caused a significant decrease in leptin while a significant increase in the levels of adiponectin in adults. Many studies support these findings (20, 33, 35, 36, 46). Our findings have important implications for understanding the effects of lifestyle modification on leptin regulation and obesity. Leptin is a hormone secreted by adipose tissue and acts centrally in the hypothalamus to regulate energy balance and appetite (48). Higher levels of leptin indicate that the hypothalamus has sufficient fat stores to suppress appetite and increase energy expenditure. Individuals with obesity typically have chronically elevated leptin levels, indicating leptin resistance, in which increased leptin signaling does not translate into appropriate appetite suppressant effects (49). The reduction in leptin observed with dietary and exercise training interventions may contribute to sensitizing the brain to the effects of leptin and better body mass control.

Several mechanisms may underlie the leptin-lowering effects of exercise training. For instance, exercise training has been shown to increase catecholamines such as epinephrine and norepinephrine, which inhibit the production and secretion of leptin in adipose tissue (50). Exercise-induced activation of the sympathetic nervous system can also directly inhibit transcription (51). In addition, the anti-inflammatory effects of exercise training may reduce leptin production, as inflammatory cytokines stimulate leptin secretion (52).

PBD are lower in fat and higher in fiber than omnivorous diets. Inadequate dietary fat consumption significantly contributes to the reduction of circulating leptin, given that acute increases in adipose triglycerides and plasma fatty acids modulate leptin secretion (53). Fiber has also been shown to bind leptin in the small intestine and inhibit its absorption. Additionally, lower amounts of protein in PBD may affect leptin levels, as protein strongly stimulates leptin secretion (22, 54).

The synergistic effect of physical activity and PBD on leptin reduction may be explained by the reduction of fat cell size and total body fat mass (39). Exercise training and negative energy balance activate triglycerides stored in fat cells and reduce fat diameter. The mass of leptin-producing adipose tissue also decreases with body mass loss caused by diet and exercise training. These morphological changes in fat probably underlie the concomitant decrease in plasma leptin levels (55).

These findings have clinical implications for the treatment of obesity. Increased leptin levels are associated with an increased risk of metabolic diseases, cardiovascular events, and some cancers (56). The leptin-lowering effects of aerobic exercise and PBD demonstrated in this meta-analysis may contribute to the reduction in morbidity and mortality observed with these lifestyle changes. For adults with obesity, combining regular aerobic exercise with a Mediterranean diet may be an effective strategy not only for body mass loss but also for improving leptin sensitivity and metabolic health (46).

The results of the current meta-analyses also showed that a combination of PBD with exercise training led to remarkable increases in adiponectin levels in adults. Other studies also support these findings (33, 34, 37, 45, 47). Several mechanisms may contribute to this upregulation of adiponectin by exercise training and PBD. First, exercise training is a potent stimulus for increased adiponectin production and release from adipose tissue. Skeletal muscle contractions during aerobic exercise, such as walking, running, or cycling, stimulate the release of adiponectin into the circulation (57). This may be mediated by the activation of p38 mitogen-activated protein kinase (MAPK) signaling in adipocytes in response to muscle contraction (58). In addition, exercise training reduces inflammation within visceral fat, which may reduce transcriptional and translational repression of adiponectin (59).

Second, PBD are rich in nutrients and bioactive compounds that have been shown to increase adiponectin levels. Specifically, high fiber intake from whole grains, fruits, vegetables, and legumes regulates adiponectin by increasing insulin sensitivity and suppressing inflammatory signals (60). PBD also consist of adequate vitamin C, magnesium, and omega-3 fatty acids from plant sources, all of which are associated with increased adiponectin production (22). Eliminating meat in Mediterranean diets may help by removing pro-inflammatory factors and improving oxidative balance (46).

Together, these synergistic mechanisms induced by aerobic exercise and PBD significantly increase adiponectin levels. The large effect size shown in this meta-analysis is clinically significant, as higher circulating adiponectin is independently associated with a variety of health benefits. Prospective studies show that increased adiponectin reduces the risk of obesity, T2DM, CVD, and some cancers (61). Adiponectin exerts these protective effects through its insulin-sensitizing, anti-inflammatory, and anti-atherogenic properties (62).

Given the increasing prevalence of hypoadiponectinemia in many populations, our findings have important implications. Adiponectin levels have declined significantly over the past few decades, likely due to increased levels of physical inactivity and unhealthy diets high in saturated fat and processed foods (63). Low adiponectin is now thought to be an independent risk factor for insulin resistance, metabolic syndrome, and CVD (64). Therefore, lifestyle modification aimed at increasing adiponectin may provide a powerful therapeutic strategy for the prevention and management of several interrelated chronic diseases.

In summary, the present meta-analysis study showed that a combination of exercise training and PBD decreased circulating leptin levels while increasing adiponectin. The decrease in leptin and increase in adiponectin may be attributed to the decrease in fat mass, as well as the direct effect of exercise training and diet composition on leptin production and secretion. Increasing chronic adiponectin and leptin levels through lifestyle modification may improve leptin sensitivity and obesity-related diseases. Further research is needed to identify long-term clinical effects and determine definitive strategies.

Overall, findings are different regarding the effect of PBD and physical activity on leptin and adiponectin levels. One study showed that PBD had no effect on leptin and adiponectin levels (21), while the other study showed that a short-term intervention with a vegetarian diet resulted in improved plasma concentrations of adiponectin and leptin (23). Certain dietary components that are lower in PBD diets, such as energy intake, saturated fat, heme iron, and red and processed meat, may influence the risk of metabolic syndrome. In addition, PBD contain more fruits, vegetables, and fiber, which protect against metabolic syndrome. Additionally, intensive lifestyle changes, including PBD diet, have been associated with a reduced risk of metabolic syndrome (65).

This meta-analysis has several strengths that lend credence to the results. To the best of our knowledge, the current study is the most comprehensive meta-analysis addressing the effects of PBD combined with exercise training on leptin and adiponectin levels in adults with or without chronic diseases. We minimized the potential for bias in the review process by executing a comprehensive search of the literature and also conducting a systematic review and reporting the meta-analytic results according to the PRISMA guidelines. Nevertheless, some potential limitations should be considered when interpreting these findings. First, diet and exercise programs varied somewhat across trials, which may introduce heterogeneity. Additional standardized intervention studies in different populations could further substantiate the effects on adiponectin. Also, some studies were conducted on healthy people and some on adults with overweight and obesity, which may distort the homogeneity of the results to some extent. One of the reasons for the high heterogeneity can be attributed to the different health characteristics of participants, such as menopause, metabolic syndrome, obstructive sleep apnea, obesity, or coronary artery disease, and the duration of the interventions. Conducting meta-regressions could potentially offer more robust findings in this case. However, the limited number of studies that provided data on BFP and BMI precluded us from conducting meta-regression or subgroup analyses to examine the relationship between leptin and adiponectin with BFP or BMI. Also, due to the small number of studies, it was not possible to perform subgroup analysis based on the type, duration, and intensity of exercise.

## 5 Conclusion

The findings of the present systematic review and meta-analysis show the important role of PBD combined with exercise training in improving leptin and adiponectin. Exercise training combined with PBD are suggested as non-invasive interventions for reducing leptin and increasing adiponectin levels to control body mass and other disorders related to obesity in adults with or without chronic diseases.

## Data Availability

The original contributions presented in the study are included in the article/Supplementary material, further inquiries can be directed to the corresponding author.

## References

[1] Caballero B. The global epidemic of obesity: an overview. Epidemiol Rev. (2007) 29:1–5. doi: 10.1093/epirev/mxm012 17569676

[2] Hruby A Hu FB. The epidemiology of obesity: a big picture. PharmacoEconomics. (2015) 33:673–89. doi: 10.1007/s40273-014-0243-x, PMID: 25471927 PMC4859313

[3] Tremblay A Clinchamps M Pereira B Courteix D Lesourd B Chapier R . Dietary fibres and the management of obesity and metabolic syndrome: the RESOLVE study. Nutrients. (2020) 12:2911. doi: 10.3390/nu12102911, PMID: 32977595 PMC7650763

[4] Khanna D Khanna S Khanna P Kahar P Patel BM. Obesity: a chronic low-grade inflammation and its markers. Cureus. (2022):14. doi: 10.7759/cureus.22711 PMC8967417 35386146

[5] Clemente-Suárez VJ Redondo-Flórez L Beltrán-Velasco AI Martín-Rodríguez A Martínez-Guardado I Navarro-Jiménez E . The role of Adipokines in health and disease. Biomedicines. (2023) 11:1290. doi: 10.3390/biomedicines11051290 37238961 PMC10216288

[6] Nikolettos K Nikolettos N Vlahos N Pagonopoulou O Asimakopoulos B. Role of leptin, adiponectin, and kisspeptin in polycystic ovarian syndrome pathogenesis. Minerva Obstet Gynecol. (2023) 75:460–7. doi: 10.23736/S2724-606X.22.05139-9, PMID: 36255161

[7] Li Y Zheng H Yang J Zhang B Xing X Zhang Z . Association of genetic variants in leptin, leptin receptor and adiponectin with hypertension risk and circulating leptin/adiponectin changes. Gene. (2023) 853:147080. doi: 10.1016/j.gene.2022.147080, PMID: 36470480

[8] Anagnostoulis S Karayiannakis AJ Lambropoulou M Efthimiadou A Polychronidis A Simopoulos C. Human leptin induces angiogenesis in vivo. Cytokine. (2008) 42:353–7. doi: 10.1016/j.cyto.2008.03.009 18448353

[9] Beltowski J. Role of leptin in blood pressure regulation and arterial hypertension. J Hypertens. (2006) 24:789–801. doi: 10.1097/01.hjh.0000222743.06584.66 16612235

[10] Minokoshi Y Toda C Okamoto S. Regulatory role of leptin in glucose and lipid metabolism in skeletal muscle. Indian J Endocrinol Metab. (2012) 16:S562–S8. doi: 10.4103/2230-8210.105573 23565491 PMC3602985

[11] Turner RT Kalra SP Wong CP Philbrick KA Lindenmaier LB Boghossian S . Peripheral leptin regulates bone formation. J Bone Miner Res. (2013) 28:22–34. doi: 10.1002/jbmr.1734, PMID: 22887758 PMC3527690

[12] Bernotiene E Palmer G Gabay C. The role of leptin in innate and adaptive immune responses. Arthritis Res. (2006) 8:217–10. doi: 10.1186/ar2004, PMID: 16879738 PMC1779438

[13] Ye Y Wu P Wang Y Yang X Ye Y Yuan J . Adiponectin, leptin, and leptin/adiponectin ratio with risk of gestational diabetes mellitus: a prospective nested case-control study among Chinese women. Diabetes Res Clin Pract. (2022) 191:110039. doi: 10.1016/j.diabres.2022.110039, PMID: 35985429

[14] Mohammadi S Arefhosseini SR Ebrahimi-Mamaeghani M Fallah P Bazi Z. Adiponectin as a potential biomarker of vascular disease. Vasc Health Risk Manag. (2015) 11:55–70. doi: 10.2147/VHRM.S48753, PMID: 25653535 PMC4303398

[15] Spranger J Kroke A Möhlig M Bergmann MM Ristow M Boeing H . Adiponectin and protection against type 2 diabetes mellitus. Lancet. (2003) 361:226–8. doi: 10.1016/S0140-6736(03)12255-6 12547549

[16] Tate CM Geliebter A. Intragastric balloon treatment for obesity: review of recent studies. Adv Ther. (2017) 34:1859–75. doi: 10.1007/s12325-017-0562-3, PMID: 28707286

[17] Wolfe BM Kvach E Eckel RH. Treatment of obesity: weight loss and bariatric surgery. Circ Res. (2016) 118:1844–55. doi: 10.1161/CIRCRESAHA.116.307591, PMID: 27230645 PMC4888907

[18] Haghighat N Ashtary-Larky D Bagheri R Aghakhani L Asbaghi O Amini M . Preservation of fat-free mass in the first year after bariatric surgery: a systematic review and meta-analysis of 122 studies and 10,758 participants. Surg Obes Relat Dis. (2022) 18:964–82. doi: 10.1016/j.soard.2022.02.022 35581110

[19] Chang S-L Nfor ON Ho C-C Lee K-J Lu W-Y Lung C-C . Combination of exercise and vegetarian diet: relationship with high density-lipoprotein cholesterol in Taiwanese adults based on MTHFR rs1801133 polymorphism. Nutrients. (2020) 12:1564. doi: 10.3390/nu12061564, PMID: 32471241 PMC7352486

[20] Telles S Naveen VK Balkrishna A Kumar S. Short term health impact of a yoga and diet change program on obesity. Med Sci Monit. (2010) 16:Cr35–40. PMID: 20037492

[21] Eichelmann F Schwingshackl L Fedirko V Aleksandrova K. Effect of plant-based diets on obesity-related inflammatory profiles: a systematic review and meta-analysis of intervention trials. Obes Rev. (2016) 17:1067–79. doi: 10.1111/obr.12439, PMID: 27405372

[22] Gogga P Janczy A Szupryczyńska N Śliwińska A Kochan Z Malgorzewicz S. Plant-based diets contribute to lower circulating leptin in healthy subjects independently of BMI. Acta Biochim Pol. (2022) 69:879–82. doi: 10.18388/abp.2020_6388, PMID: 36269890

[23] Lederer A-K Storz MA Huber R Hannibal L Neumann E. Plasma leptin and adiponectin after a 4-week vegan diet: a randomized-controlled pilot trial in healthy participants. Int J Environ Res Public Health. (2022) 19:11370. doi: 10.3390/ijerph191811370, PMID: 36141644 PMC9517500

[24] Menzel J Biemann R Longree A Isermann B Mai K Schulze MB . Associations of a vegan diet with inflammatory biomarkers. Sci Rep. (2020) 10:1933. doi: 10.1038/s41598-020-58875-x, PMID: 32029816 PMC7005174

[25] Hargreaves SM Rosenfeld DL Moreira AVB Zandonadi RP. Plant-based and vegetarian diets: an overview and definition of these dietary patterns. Eur J Nutr. (2023) 62:1109–21. doi: 10.1007/s00394-023-03086-z, PMID: 36681744

[26] Craig WJ Mangels AR Fresán U Marsh K Miles FL Saunders AV . The safe and effective use of plant-based diets with guidelines for health professionals. Nutrients. (2021) 13:4144. doi: 10.3390/nu13114144, PMID: 34836399 PMC8623061

[27] Nurnazahiah A Lua PL Shahril MR. Adiponectin, leptin and objectively measured physical activity in adults: a narrative review. Malays J Med Sci. (2016) 23:7–24. doi: 10.21315/mjms2016.23.6.2, PMID: 28090175 PMC5181988

[28] Eskandari M Hooshmand Moghadam B Bagheri R Ashtary-Larky D Eskandari E Nordvall M . Effects of interval jump rope exercise combined with dark chocolate supplementation on inflammatory adipokine, cytokine concentrations, and body composition in obese adolescent boys. Nutrients. (2020) 12:3011. doi: 10.3390/nu12103011, PMID: 33007981 PMC7600985

[29] Jadhav RA Maiya GA Hombali A Umakanth S Shivashankar K. Effect of physical activity promotion on adiponectin, leptin and other inflammatory markers in prediabetes: a systematic review and meta-analysis of randomized controlled trials. Acta Diabetol. (2021) 58:419–29. doi: 10.1007/s00592-020-01626-1, PMID: 33211181 PMC8053655

[30] Becic T Studenik C Hoffmann G. Exercise increases adiponectin and reduces leptin levels in prediabetic and diabetic individuals: systematic review and meta-analysis of randomized controlled trials. Med Sci. (2018) 6:97. doi: 10.3390/medsci6040097 PMC6318757 30380802

[31] Tuso PJ Ismail MH Ha BP Bartolotto C. Nutritional update for physicians: plant-based diets. Perm J. (2013) 17:61–6. doi: 10.7812/TPP/12-085, PMID: 23704846 PMC3662288

[32] Li H Zeng X Wang Y Zhang Z Zhu Y Li X . A prospective study of healthful and unhealthful plant-based diet and risk of overall and cause-specific mortality. Eur J Nutr. (2022) 61:387–98. doi: 10.1007/s00394-021-02660-7, PMID: 34379193

[33] Bendinelli B Masala G Bella CD Assedi M Benagiano M Pratesi S . Adipocytokine plasma level changes in a 24-month dietary and physical activity randomised intervention trial in postmenopausal women. Eur J Nutr. (2023) 62:1185–94. doi: 10.1007/s00394-022-03055-y, PMID: 36454365

[34] Esposito K di Palo C Maiorino MI Petrizzo M Bellastella G Siniscalchi I . Long-term effect of Mediterranean-style diet and calorie restriction on biomarkers of longevity and oxidative stress in overweight men. Cardiol Res Pract. (2011) 2011:293916:1–5. doi: 10.4061/2011/293916, PMID: 21197397 PMC3010676

[35] Voeghtly LM Neatrour DM Decewicz DJ Burke A Haberkorn MJ Lechak F . Cardiometabolic risk reduction in an intensive cardiovascular health program. Nutr Metab Cardiovasc Dis. (2013) 23:662–9. doi: 10.1016/j.numecd.2012.01.012 22633795

[36] Salas-Salvadó J Díaz-López A Ruiz-Canela M Basora J Fitó M Corella D . Effect of a lifestyle intervention program with energy-restricted Mediterranean diet and exercise on weight loss and cardiovascular risk factors: one-year results of the PREDIMED-plus trial. Diabetes Care. (2019) 42:777–88. doi: 10.2337/dc18-0836, PMID: 30389673

[37] Georgoulis M Yiannakouris N Tenta R Fragopoulou E Kechribari I Lamprou K . A weight-loss Mediterranean diet/lifestyle intervention ameliorates inflammation and oxidative stress in patients with obstructive sleep apnea: results of the "MIMOSA" randomized clinical trial. Eur J Nutr. (2021) 60:3799–810. doi: 10.1007/s00394-021-02552-w, PMID: 33839919

[38] Moher D Liberati A Tetzlaff J Altman DG Group* P. Preferred reporting items for systematic reviews and meta-analyses: the PRISMA statement. Ann Intern Med. (2009) 151:264–9. doi: 10.7326/0003-4819-151-4-200908180-00135 19622511

[39] Long Y Ye H Yang J Tao X Xie H Zhang J . Effects of a vegetarian diet combined with aerobic exercise on glycemic control, insulin resistance, and body composition: a systematic review and meta-analysis. Eat Weight Disord. (2023) 28:9. doi: 10.1007/s40519-023-01536-5 36790517 PMC9931794

[40] Niu Y Cao H Zhou H Cao J Wang Z. Effects of a vegetarian diet combined with exercise on lipid profiles and blood pressure: a systematic review and meta-analysis. Crit Rev Food Sci Nutr. (2022) 64:2289–2303. doi: 10.1080/10408398.2022.2122923 36106474

[41] Wan X Wang W Liu J Tong T. Estimating the sample mean and standard deviation from the sample size, median, range and/or interquartile range. BMC Med Res Methodol. (2014) 14:1–13. doi: 10.1186/1471-2288-14-135, PMID: 25524443 PMC4383202

[42] Higgins JP. Cochrane handbook for systematic reviews of interventions version 5.0. 1 The Cochrane Collaboration (2008).

[43] Hozo SP Djulbegovic B Hozo I. Estimating the mean and variance from the median, range, and the size of a sample. BMC Med Res Methodol. (2005) 5:1–10. doi: 10.1186/1471-2288-5-13, PMID: 15840177 PMC1097734

[44] Egger M Smith GD Schneider M Minder C. Bias in meta-analysis detected by a simple, graphical test. BMJ. (1997) 315:629–34. doi: 10.1136/bmj.315.7109.629, PMID: 9310563 PMC2127453

[45] Kahleova H Matoulek M Malinska H Oliyarnik O Kazdova L Neskudla T . Vegetarian diet improves insulin resistance and oxidative stress markers more than conventional diet in subjects with type 2 diabetes. Diabet Med. (2011) 28:549–59. doi: 10.1111/j.1464-5491.2010.03209.x, PMID: 21480966 PMC3427880

[46] Hernando-Redondo J Toloba A Benaiges D Salas-Salvadó J Martínez-Gonzalez MA Corella D . Mid- and long-term changes in satiety-related hormones, lipid and glucose metabolism, and inflammation after a Mediterranean diet intervention with the goal of losing weight: a randomized, clinical trial. Front Nutr. (2022) 9:950900. doi: 10.3389/fnut.2022.950900 36466401 PMC9716624

[47] Koeder C Anand C Husain S Kranz RM Schoch N Alzughayyar D . Exploratory analysis of the effect of a controlled lifestyle intervention on inflammatory markers – the healthy lifestyle community Programme (cohort 2). BMC Nutr. (2023) 9:25. doi: 10.1186/s40795-023-00684-2, PMID: 36747285 PMC9900566

[48] Friedman JM Halaas JL. Leptin and the regulation of body weight in mammals. Nature. (1998) 395:763–70. doi: 10.1038/27376 9796811

[49] Myers MG Jr Leibel RL Seeley RJ Schwartz MW. Obesity and leptin resistance: distinguishing cause from effect. Trends Endocrinol Metab. (2010) 21:643–51. doi: 10.1016/j.tem.2010.08.002, PMID: 20846876 PMC2967652

[50] Essig DA Alderson NL Ferguson MA Bartoli WP Durstine JL. Delayed effects of exercise on the plasma leptin concentration. Metabolism. (2000) 49:395–9. doi: 10.1016/S0026-0495(00)90396-2, PMID: 10726920

[51] Daniela M Catalina L Ilie O Paula M Daniel-Andrei I Ioana B. Effects of exercise training on the autonomic nervous system with a focus on anti-inflammatory and antioxidants effects. Antioxidants (Basel). (2022) 11:350. doi: 10.3390/antiox11020350, PMID: 35204231 PMC8868289

[52] Pérez-Pérez A Sánchez-Jiménez F Vilariño-García T Sánchez-Margalet V. Role of leptin in inflammation and vice versa. Int J Mol Sci. (2020) 21:5887. doi: 10.3390/ijms21165887 32824322 PMC7460646

[53] Izadi V Saraf-Bank S Azadbakht L. Dietary intakes and leptin concentrations. ARYA Atheroscler. (2014) 10:266–72. PMID: 25477984 PMC4251481

[54] Kim MH Kim H. Role of leptin in the digestive system. Front Pharmacol. (2021) 12:660040. doi: 10.3389/fphar.2021.660040, PMID: 33935782 PMC8086408

[55] Lin D Sturgeon KM Gordon BR Brown JC Sears DD Sarwer DB . WISER survivor trial: combined effect of exercise and weight loss interventions on adiponectin and leptin levels in breast Cancer survivors with overweight or obesity. Nutrients. (2023) 15:3453. doi: 10.3390/nu15153453, PMID: 37571390 PMC10421485

[56] Khanbabaei N Mozafar Saadati H Valizadeh Shahbazloo S Hoseinpoor R Naderi SH Taghvamanesh R . Association of serum leptin with angiographically proven cardiovascular disease and with components of the metabolic syndrome: a cross-sectional study in East Azerbaijan. Cardiovasc Endocrinol Metab. (2021) 10:45–50. doi: 10.1097/XCE.0000000000000227, PMID: 33634255 PMC7901824

[57] Simpson KA Singh MAF. Effects of exercise on adiponectin: a systematic review. Obesity. (2008) 16:241–56. doi: 10.1038/oby.2007.53 18239630

[58] Qiao L Kinney B Yoo H s Lee B Schaack J Shao J. Adiponectin increases skeletal muscle mitochondrial biogenesis by suppressing mitogen-activated protein kinase phosphatase-1. Diabetes. (2012) 61:1463–70. doi: 10.2337/db11-1475, PMID: 22415879 PMC3357265

[59] You T Berman DM Ryan AS Nicklas BJ. Effects of hypocaloric diet and exercise training on inflammation and adipocyte lipolysis in obese postmenopausal women. J Clin Endocrinol Metab. (2004) 89:1739–46. doi: 10.1210/jc.2003-031310, PMID: 15070939

[60] Neyrinck AM Van Hee VF Piront N De Backer F Toussaint O Cani PD . Wheat-derived arabinoxylan oligosaccharides with prebiotic effect increase satietogenic gut peptides and reduce metabolic endotoxemia in diet-induced obese mice. *Nutr*. Diabetes. (2012) 2:e28. doi: 10.1038/nutd.2011.24, PMID: 23154683 PMC3302144

[61] Khoramipour K Chamari K Hekmatikar AA Ziyaiyan A Taherkhani S Elguindy NM . Adiponectin: structure, physiological functions, role in diseases, and effects of nutrition. Nutrients. (2021) 13:1180. doi: 10.3390/nu13041180, PMID: 33918360 PMC8066826

[62] Oh DK Ciaraldi T Henry RR. Adiponectin in health and disease. Diabetes Obes Metab. (2007) 9:282–9. doi: 10.1111/j.1463-1326.2006.00610.x 17391153

[63] Janiszewska J Ostrowska J Szostak-Węgierek D. The influence of nutrition on adiponectin-a narrative review. Nutrients. (2021) 13:1394. doi: 10.3390/nu13051394, PMID: 33919141 PMC8143119

[64] Muratsu J Kamide K Fujimoto T Takeya Y Sugimoto K Taniyama Y . The combination of high levels of adiponectin and insulin resistance are affected by aging in non-obese old peoples. Front Endocrinol. (2022) 12:805244. doi: 10.3389/fendo.2021.805244 PMC8777034 35069451

[65] Turner-McGrievy G Harris M. Key elements of plant-based diets associated with reduced risk of metabolic syndrome. Curr Diab Rep. (2014) 14:524–9. doi: 10.1007/s11892-014-0524-y, PMID: 25084991

