# Supplementary material for: Effects of plant-based diets combined with exercise training on leptin and adiponectin levels in adults with or without chronic diseases: a systematic review and meta-analysis of clinical studies
Source: Front Nutr. 2024 Oct 9;11:1465378. doi: 10.3389/fnut.2024.1465378 (PMC11496297; doi:10.3389/fnut.2024.1465378)
Supplement: Supplementary file 1 [file Table_1.docx]

**Supplementary Table 1.** Search strategy

| **Databases** | **Search strategy** | **Limits** | **Results** |
| --- | --- | --- | --- |
| PubMed | (("diet" or "Nordic diet" or "Mediterranean diet" or "vegetarian diet" or "plant-based diet" or "Paleolithic diet" or "dietary pattern" or "DASH" or "vegan diet" or "lacto-ovo-vegetarian diet" or "vegetarian diet") AND ("Exercise" or "training" or "Exercise training" or "Physical Activity")) AND ("adipokine" "adipocytokine" or "adiponectin" or "leptin") | Humans, English | 808 |
| Scopus | (("diet" or "Nordic diet" or "Mediterranean diet" or "vegetarian diet" or "plant-based diet" or "Paleolithic diet" or "dietary pattern" or "DASH" or "vegan diet" or "lacto-ovo-vegetarian diet" or "vegetarian diet") AND ("Exercise" or "training" or "Exercise training" or "Physical Activity")) AND ("adipokine" "adipocytokine" or "adiponectin" or "leptin") | Article, English | 136 |
| Web of science | (("diet" or "Nordic diet" or "Mediterranean diet" or "vegetarian diet" or "plant-based diet" or "Paleolithic diet" or "dietary pattern" or "DASH" or "vegan diet" or "lacto-ovo-vegetarian diet" or "vegetarian diet") AND ("Exercise" or "training" or "Exercise training" or "Physical Activity")) AND ("adipokine" "adipocytokine" or "adiponectin" or "leptin") | Article, English | 1465 |
