# Supplementary material for: Effects of plant-based diets combined with exercise training on leptin and adiponectin levels in adults with or without chronic diseases: a systematic review and meta-analysis of clinical studies
Source: Front Nutr. 2024 Oct 9;11:1465378. doi: 10.3389/fnut.2024.1465378 (PMC11496297; doi:10.3389/fnut.2024.1465378)
Supplement: Supplementary file 2 [file Table_2.docx]

**Supplementary Table 2**. Risk of bias assessment (PEDro scale)

| **Authors and Year of Publication** | **1** | **2** | **3** | **4** | **5** | **6** | **7** | **8** | **Total score** |
| --- | --- | --- | --- | --- | --- | --- | --- | --- | --- |
| Bendinelli et al. 2023 |  |  | × |  |  |  |  |  | 7 |
| Esposito et al. 2011 |  |  | × | × |  | × |  |  | 5 |
| Georgoulis et al. 2021 |  |  |  |  |  |  |  |  | 8 |
| Hernando-Redondo et al. 2022 |  |  | × | × |  | × |  |  | 5 |
| Salas-Salvadó et al. 2019 |  |  | × | × |  |  |  |  | 6 |
| Kahleova et al. 2011 |  |  | × | × |  |  |  |  | 6 |
| Koeder et al. 2023 |  | × | × | × | × | × |  |  | 3 |
| Telles et al. 2010 |  | × | × | × |  | × |  |  | 4 |
| Voeghtly et al. 2013 |  | × | × | × |  |  |  |  | 5 |

1. specified eligibility criteria, (2) randomized participant allocation, (3) concealed allocation, (4) blinding of all assessors, (5) evaluated outcomes in 85% of participants, (6) intention-to-treat (ITT) analysis, (7) reporting of statistical comparisons between groups, (8) and point measures and measures of variability.
